# Global gene expression responses of Atlantic salmon skin to *Moritella viscosa*

**DOI:** 10.1038/s41598-022-08341-7

**Published:** 2022-03-17

**Authors:** Khalil Eslamloo, Surendra Kumar, Xi Xue, Kathleen S. Parrish, Sara L. Purcell, Mark D. Fast, Matthew L. Rise

**Affiliations:** 1 Department of Ocean Sciences, Memorial University of Newfoundland, St. John’s, NL Canada; 2 Hoplite Laboratory, Department of Pathology and Microbiology, Atlantic Veterinary College, University of Prince Edward Island, Charlottetown, PEI, Canada

**Keywords:** Bacterial infection, Transcriptomics

## Abstract

*Moritella viscosa* is a Gram-negative pathogen that causes large, chronic ulcers, known as winter-ulcer disease, in the skin of several fish species including Atlantic salmon. We used a bath challenge approach to profile the transcriptome responses of *M. viscosa*-infected Atlantic salmon skin at the lesion (Mv-At) and away from the lesion (Mv-Aw) sites. *M. viscosa* infection was confirmed through RNA-based qPCR assays. RNA-Seq identified 5212 and 2911 transcripts differentially expressed in the Mv-At compared to no-infection control and Mv-Aw groups, respectively. Also, there were 563 differentially expressed transcripts when comparing the Mv-Aw to control samples. Our results suggest that *M. viscosa* caused massive and strong, but largely infection site-focused, transcriptome dysregulations in Atlantic salmon skin, and its effects beyond the skin lesion site were comparably subtle. The *M. viscosa*-induced transcripts of Atlantic salmon were mainly involved in innate and adaptive immune response-related pathways, whereas the suppressed transcripts by this pathogen were largely connected to developmental and cellular processes. As validated by qPCR, *M. viscosa* dysregulated transcripts encoding receptors, signal transducers, transcription factors and immune effectors playing roles in TLR- and IFN-dependent pathways as well as immunoregulation, antigen presentation and T-cell development. This study broadened the current understanding of molecular pathways underlying *M. viscosa*-triggered responses of Atlantic salmon, and identified biomarkers that may assist to diagnose and combat this pathogen.

## Introduction

Due to the growing human population as well as the over-exploitation and collapse of many marine resources, aquaculture is becoming the main source fulfilling the future global demand for seafood^1,2^. Among aquaculture species, Atlantic salmon (*Salmo salar*) is extensively farmed in several countries such as Canada, Norway and Chile, and it is known as the most economically important species in marine finfish aquaculture^2,3^. Atlantic salmon farming encounters several health issues such as bacterial pathogens that can cause economic losses^4,5^. Understanding the molecular mechanisms underlying Atlantic salmon response to bacterial disease is pivotal to developing appropriate strategies for combating these issues in intensive farming situations. In addition, salmonids have autotetraploid genomes as a result of a salmonid-specific whole-genome duplication event (Ss4R), occurring approximately 80 million years ago in their common ancestor^6^. Atlantic salmon has a well-characterised genome, which shares a high degree of similarity to other salmonids^6^; therefore, Atlantic salmon may be considered as a molecular model for studying the antibacterial responses of the members of family Salmonidae.

Gram-negative *Moritella viscosa*, previously known as *Vibrio viscosus*^7^, has been described as the causative agent of winter-ulcer disease in Atlantic salmon^8^. Winter-ulcer disease has been reported to cause mortalities, animal welfare problems and considerable financial losses in several countries such as Canada, Norway and Ireland^9^. Winter-ulcer disease is associated with low marine water temperature (i.e. below 10 °C), and causes large, chronic ulcers in Atlantic salmon skin^10^. *M. viscosa* infection was suggested to be initiated from both skin and gill of Atlantic salmon^11^, and the pathogenic mechanism of this bacterium in salmonid cells is delivered through cytoskeleton disruption, pore formation and lytic processes^12^. In addition to Atlantic salmon and rainbow trout (*Oncorhynchus mykiss*)^13^, *M. viscosa* may infect Atlantic cod (*Gadus morhua*), halibut (*Hippoglossus hippoglossus*)^14^, turbot (*Scophthalmus maximus*)^15^ and lumpfish (*Cyclopterus lumpus*)^16^.

*M. viscosa* antigen-containing vaccines have shown varying degrees of protection against winter-ulcer disease; however, new genomics-based approaches can help us better understand the interaction of this pathogen and its host to assist with management of disease^9,17^. Previous molecular-based studies have examined individual virulence factors as well as whole transcriptome and genome analysis of *M. viscosa*^18–23^. As well, in vitro studies in SHK-1 cells have shown inflammatory gene induction (i.e. *interleukin 1 beta* [*il1b*] and *il8*) following *M. viscosa*^24^ exposure, and targeted gene studies in Atlantic salmon head kidney have shown inflammatory and complement factors being induced after bath challenge with this pathogen^8^. Although a recent study used microarray analyses to identify *M. viscosa*-responsive transcripts in the spleen, head kidney and gill of Atlantic salmon^25^, a complete transcriptomic profile and understanding of the host pathways involved in response of Atlantic salmon skin to *M. viscosa* is lacking.

RNA sequencing (RNA-Seq) analyses can be employed to screen the transcriptome of immunological responses in various species, yielding discovery of responsive genes and biomarkers as well as activated signalling pathways in response to a given stimuli^26^. RNA-Seq was been successfully applied to understand fish immune mechanisms activated in response to various bacterial and viral pathogens or their derived pathogen-associated molecular patterns (PAMPs)^27^. RNA-Seq-based studies previously profiled Atlantic salmon response (e.g. head kidney) to *Piscirickettsia salmonis*^28^, and Infectious salmon anaemia virus (ISAV)^29^ as well as Atlantic salmon macrophage/dendritic-like cell line to salmon alphavirus subtype 3 (SAV-3)^30^. In the present study, we used RNA-Seq to profile the transcriptome response of Atlantic salmon skin to *M. viscosa* infection. Our study identified a large number of *M. viscosa*-responsive transcripts at the skin lesion site as well as non-lesion site (away from lesion) of the infected fish, compared with the non-infected control samples. Our findings provide a better understanding of the genes and molecular pathways involved in Atlantic salmon response to *M. viscosa*, and can be used for future studies on improving Atlantic salmon resistance to winter-ulcer disease.

## Results

### RNA-Seq and transcriptome annotation

RNA from the lesion (Mv-At: *n* = 4) and away (Mv-Aw: *n* = 4) skin samples of Atlantic salmon infected with *M. viscosa* for 29 days were used for RNA-Seq analyses. The developed lesions of infected fish, used for RNA-Seq, were identified as stage 3 lesions (i.e. dermal ulceration along with visible muscle tissue). RNA samples from the skin of 4 uninfected fish in the control group were also selected for RNA-Seq analyses (12 samples in total). The number of raw reads generated by RNA-Seq for each sample varied between ~ 40 and 87 million (Supplemental Table S1). Following the Trimmomatic filtering, ~ 96–97% of the paired-end reads in each sample survived and were used for mapping. Using StringTie, ~ 83–85% of the processed reads were uniquely mapped to the Atlantic salmon genome, whereas 6–7% of them were mapped concordantly more than one time. Both uniquely- and multi-mapped reads were used for transcript assembly of the present study. Overall, ~ 94–96% of the processed reads in each sample were mapped to the Atlantic salmon genome (Supplemental Table S1).

### *Moritella viscosa*-responsive transcripts in Atlantic salmon skin

**Figure 1:**
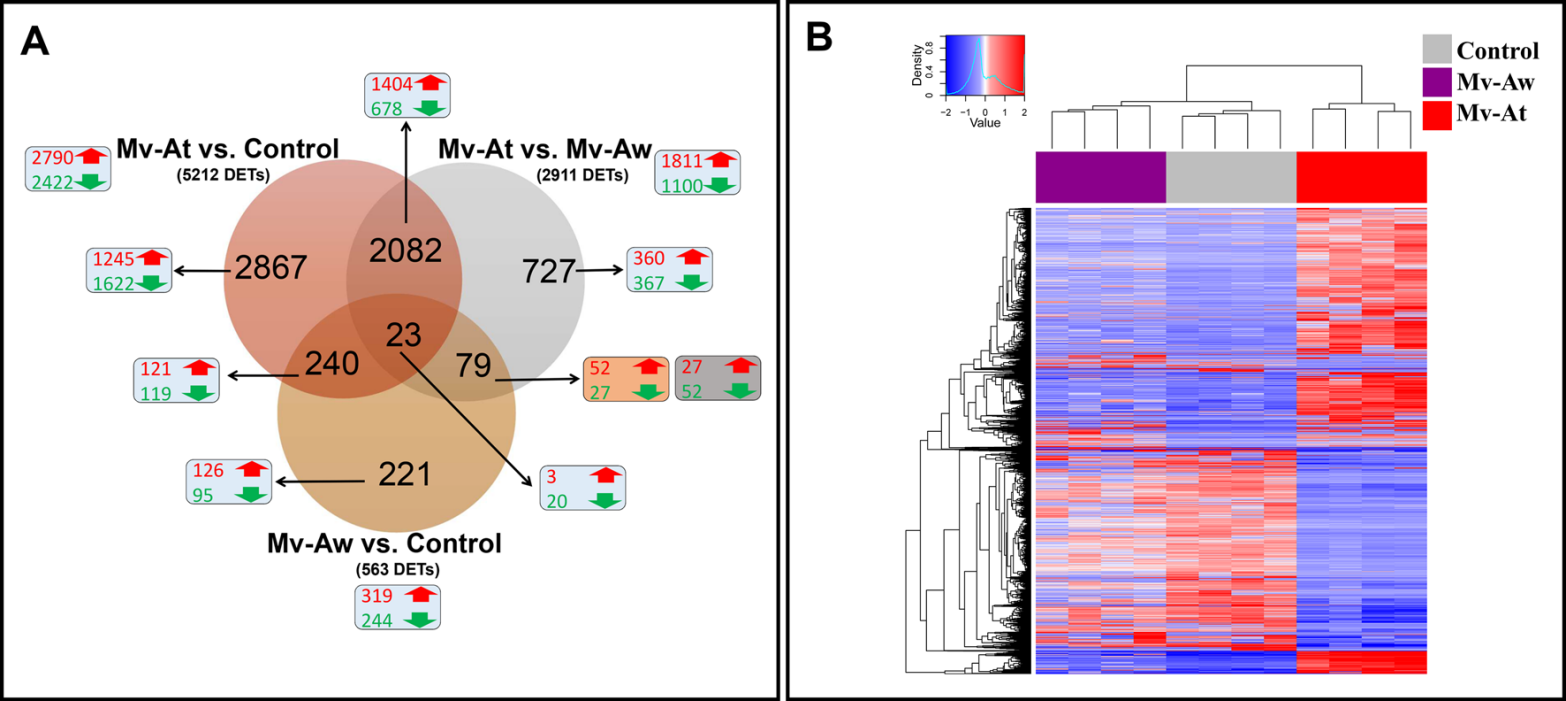
RNA-Seq results of Atlantic salmon skin response to *M. viscosa* infection. The skin samples of *M. viscosa*-infected Atlantic salmon at the lesion (Mv-At) and away (Mv-Aw) sites, as well as the skin samples of non-infected control group, were used for RNA-Seq (*n* = 4). Differentially expressed transcripts (DETs) were identified by DESeq2 (*p* adj < 0.01). (**A**) Overview of RNA-Seq results. The number of up-regulated (red) and down-regulated (green) transcripts in a given comparison is shown in the light turquoise boxes, whereas orange and grey boxes indicate DETs associated with Mv-At vs. Mv-Aw and Mv-Aw vs. Control, respectively. (**B**) Hierarchical clustering analyses of RNA-Seq results, using all DETs identified in the present study.

We used DESeq2 analysis (adjusted *p* value < 0.01) to identify the differentially-expressed transcripts (DETs) among treatments (Fig. 1A; Supplemental Table S2). There were 5212 transcripts (2790 up- and 2422 down-regulated) responsive to *M. viscosa* infection at the lesion site, compared to the control (Mv-At vs. Control). Also, 2911 transcripts (1811 up- and 1100 down-regulated) were differentially expressed (Fig. 1A; Supplemental Table S2) between the lesion and away skin sites of *M. viscosa*-infected fish (Mv-At vs. Mv-Aw). Among these *M. viscosa*-responsive transcripts, 2867 and 727 DETs were specific to Mv-At vs. Control and Mv-At vs. Mv-Aw comparisons, respectively, whereas there were 2082 DETs (1404 up- and 678 down-regulated) overlapping between both transcript lists (Fig. 1A; Supplemental Table S2). Moreover, we identified 563 DETs (319 up- and 244 down-regulated) for away skin site of *M. viscosa*-infected fish (Mv-Aw vs. Control). As shown by Venn diagram (Fig. 1A), 221 DETs were specific to the away skin site transcript list (Mv-Aw vs. Control), and 23 DETs overlapped among all comparisons. As shown in Fig. 1B, all the samples associated with a given group (i.e. Mv-At, Mv-Aw and Control) were clustered together based on the expression of all the identified DETs in this study. Supplemental Fig. S1 shows the genomic distribution of all DETs, identified herein, on Atlantic salmon chromosomes. Among genomic-annotated transcripts, the largest numbers of DETs were found to be located in the Atlantic salmon chromosomes 1 and 3, with the lowest number of DETs associated with chromosome 8 of this species. There was a significant correlation between the number of DETs placed on each Atlantic salmon chromosome and the size of each chromosome (Pearson's correlation *r* = 0.828; *p* < 0.0001).

### Pathway enrichment analyses

**Figure 2:**
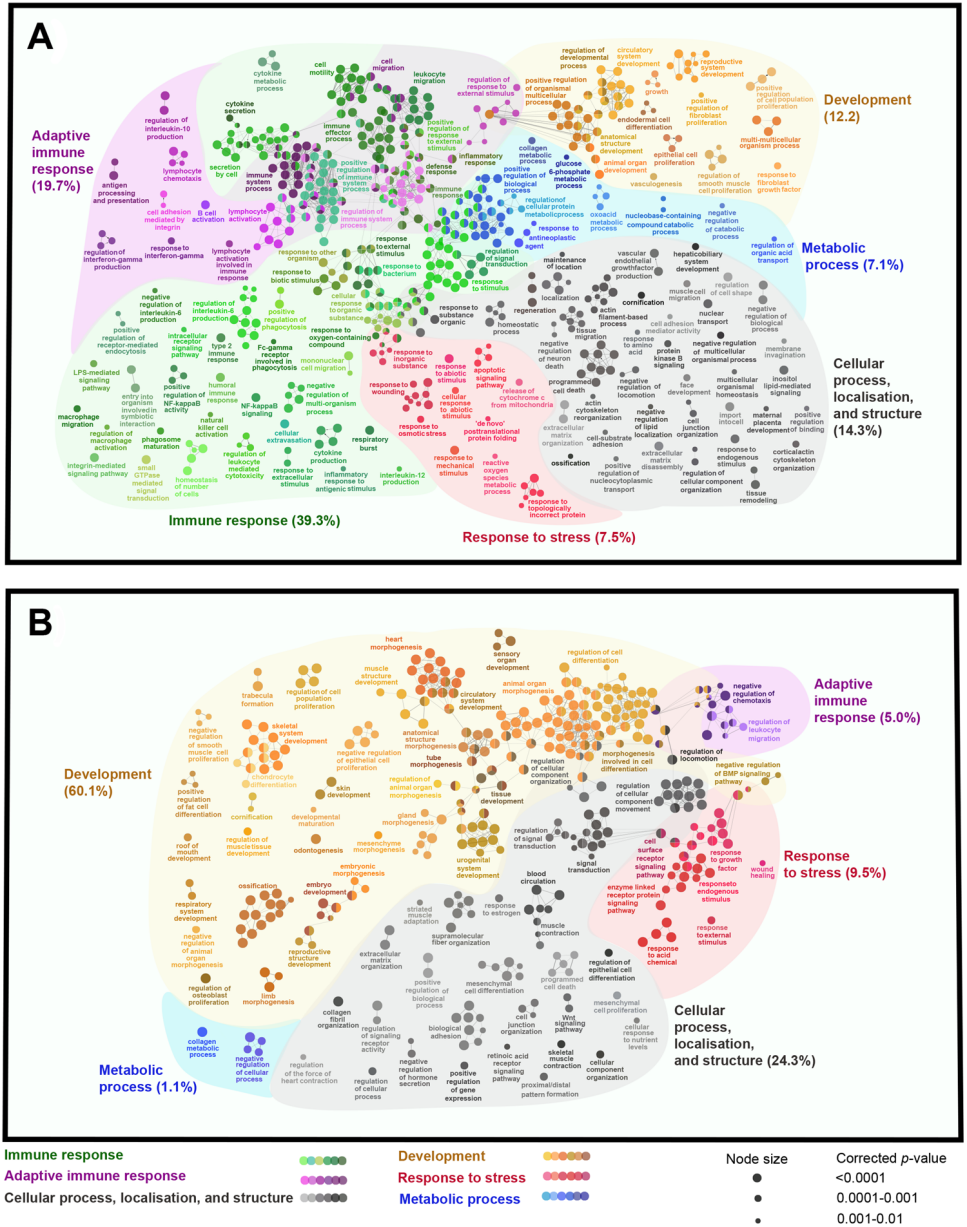
The pathways enriched in *M. viscosa*-responsive transcripts. Nodes represent Biological Process (BP) GO terms [right-sided hypergeometric test, *p* values (*p* < 0.01) corrected by Benjamini-Hochberg] significantly enriched in transcripts up- (**A**) or down-regulated (**B**) by *M. viscosa* infection at the lesion site (Mv-At vs. Control; 5212 DETs), compared to the whole RNA-Seq dataset (used as Reference). Nodes are coloured according to the functional theme to which they were assigned. Node size corresponds to different *p* value ranges (i.e. *p* < 0.0001, 0.0001–0.001, and 0.001–0.01). Highly related terms (kappa coefficient > 0.4) are connected with grey lines. Individual GO terms (i.e. single node) and GO networks (i.e. multiple connected nodes) are grouped by functional theme.

ClueGO was used to determine the Biological Processes (BPs) enriched in *M. viscosa*-responsive transcript lists compared with the entire RNA-Seq dataset (i.e. all transcripts from all 12 samples used for DESeq2 analysis). We first tested the BPs over-represented in up- and down-regulated lists of the *M. viscosa* lesion site (Mv-At vs. Control). The enriched BPs identified herein were further classified into functional themes using Gene Ontology Browser (http://www.informatics.jax.org). The 666 BPs enriched (*p* < 0.01) in transcripts up-regulated in the lesion site compared to Control were associated with immune response (39.3%), adaptive immune response (19.7%), cellular process, localisation, and structure (14.3%), development (12.2%), response to stress (7.5%) and metabolic process (7.1%) (Fig. 2A; Supplemental Table S3A). The enriched BPs associated with adaptive immune response were involved with various functions such as lymphocyte activation (e.g. T-/B-cell activation and lymphocyte differentiation), antigen presentation (e.g. antigen processing and presentation and regulation of leukocyte cell–cell adhesion) and cell migration (e.g. chemotaxis, regulation of lymphocyte migration and positive regulation of leukocyte chemotaxis). Immune response-classified BPs enriched in *M. viscosa*-induced transcripts were connected to various functions, including antibacterial response (e.g. response to bacterium and lipopolysaccharide (LPS)-mediated signalling pathway), cytokine signalling (e.g. interleukin-1 beta production, regulation of cytokine secretion and response to cytokine) and immunoregulation (e.g. regulation of innate immune response and regulation of leukocyte activation; Fig. 2A; Supplemental Table S3A). On the other hand, there were 441 BPs enriched for down-regulated transcripts at the lesion site (Mv-At vs. Control group), and the largest proportions of them were related to development (60.1%; e.g. skin/epidermis development, muscle cell proliferation, regulation of epithelial cell proliferation and neurogenesis) as well as cellular process, localisation, and structure (24.3%; e.g. apoptotic process, actin cytoskeleton organisation and regulation of localisation) (Fig. 2B; Supplemental Table S3B). There were 556 BPs enriched (*p* < 0.01) for the up-regulated transcripts in the Mv-At vs. Mv-Aw comparison, and 514 of them overlapped with the BPs over-represented in the up-regulated list of Mv-At vs. Control (Supplemental Table S3C). Further, 138 BPs were found to be over-represented in the down-regulated transcript list of Mv-At vs. Mv-Aw, 127 of which overlapped with the BPs enriched for suppressed transcripts in the Mv-At vs. Control comparison (Supplemental Table S3D).

### qPCR validation

**Figure 3:**
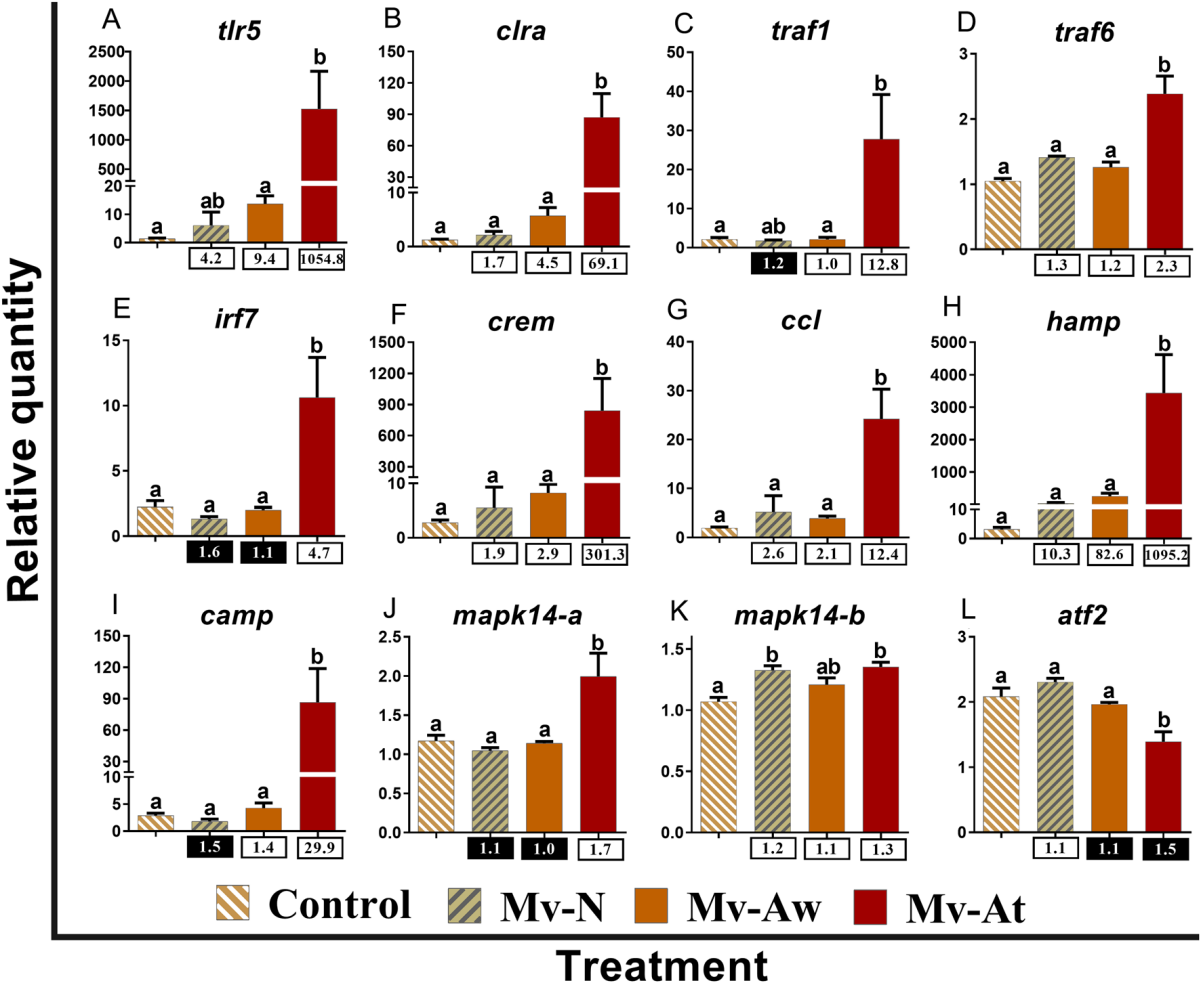
qPCR for *M. viscosa*-responsive transcripts playing putative roles in innate immune and antibacterial responses. The skin samples of *M. viscosa*-infected Atlantic salmon at the lesion (Mv-At; *n* = 4) and away (Mv-Aw; *n* = 4) sites, as well as the samples of fish with no lesion in *M. viscosa* group (Mv-N; *n* = 3) and non-infected control group (Control; *n* = 4), were used for qPCR validation. Data are presented as mean ± SE, with the lowest expressing sample as calibrator [i.e. set to relative quantity (RQ) 1.0]. Letters in each panel indicate significant differences between groups, as determined by one-way ANOVA with Tukey’s post hoc test (*p* < 0.05). Fold-changes are shown below the groups, calculated as mean RQ of the group/mean RQ of the control. Black boxes indicate the down-regulated or negative fold-changes, which were calculated as 1/fold-change for comparisons that yielded fold-change values less than 1. Gene symbols are shown at the top of each panel (**A**–**L**).

**Figure 4:**
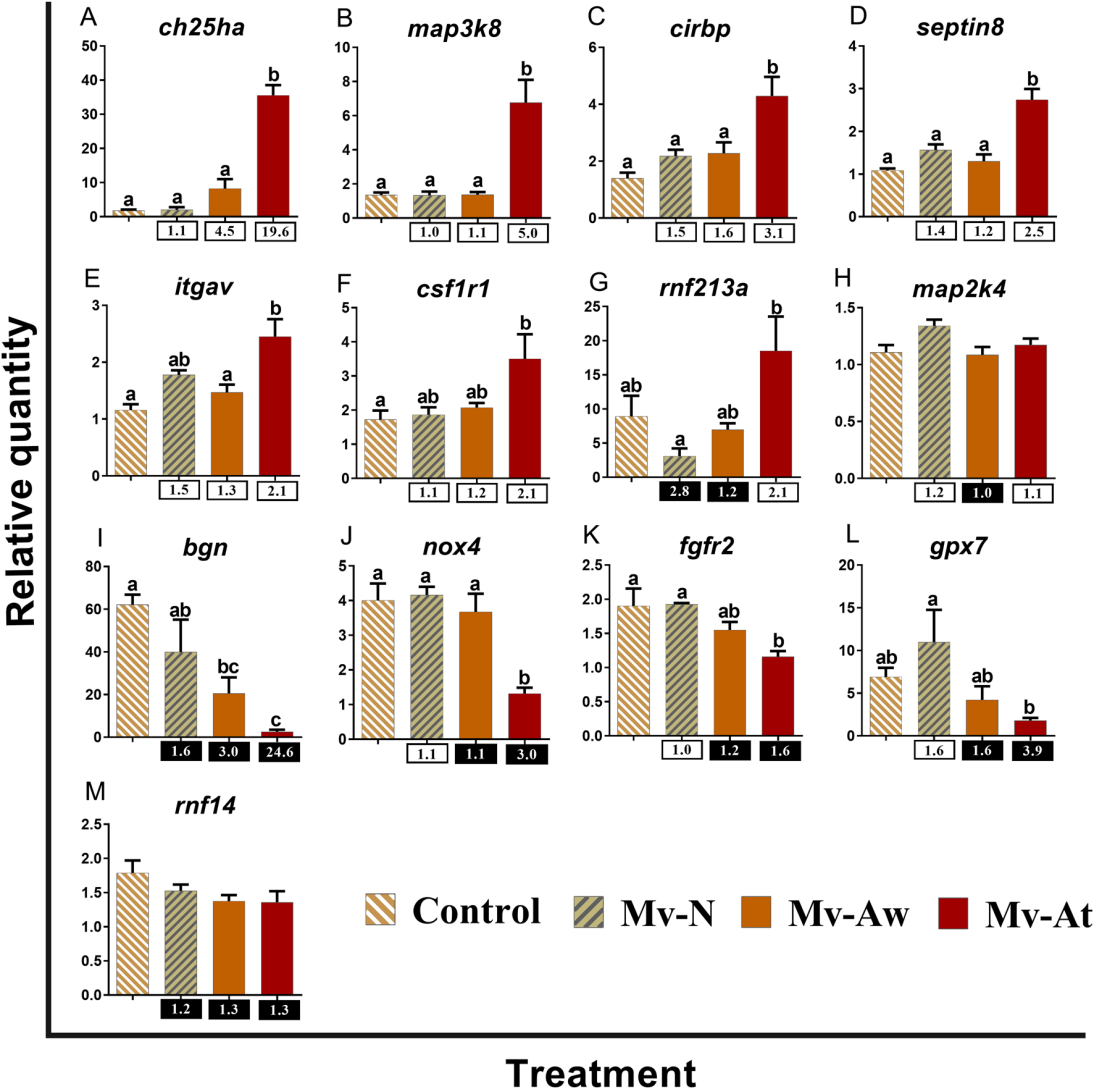
qPCR for *M. viscosa*-responsive transcripts playing putative roles in regulation of immune and inflammatory responses. The skin samples of *M. viscosa*-infected Atlantic salmon at the lesion (Mv-At; *n* = 4) and away (Mv-Aw; *n* = 4) sites, as well as the samples of fish with no lesion in *M. viscosa* group (Mv-N; *n* = 3) and non-infected control group (Control; *n* = 4), were used for qPCR validation. Data are presented as mean ± SE, with the lowest expressing sample as calibrator [i.e. set to relative quantity (RQ) 1.0]. Letters in each panel indicate significant differences between groups, as determined by one-way ANOVA with Tukey’s post hoc test (*p* < 0.05). Fold-changes are shown below the groups, calculated as mean RQ of the group/ mean RQ of the control. Black boxes indicate the down-regulated or negative fold-changes, which were calculated as 1/fold-change for comparisons that yielded fold-change values less than 1. Gene symbols are shown at the top of each panel (**A**–**M**).

**Figure 5:**
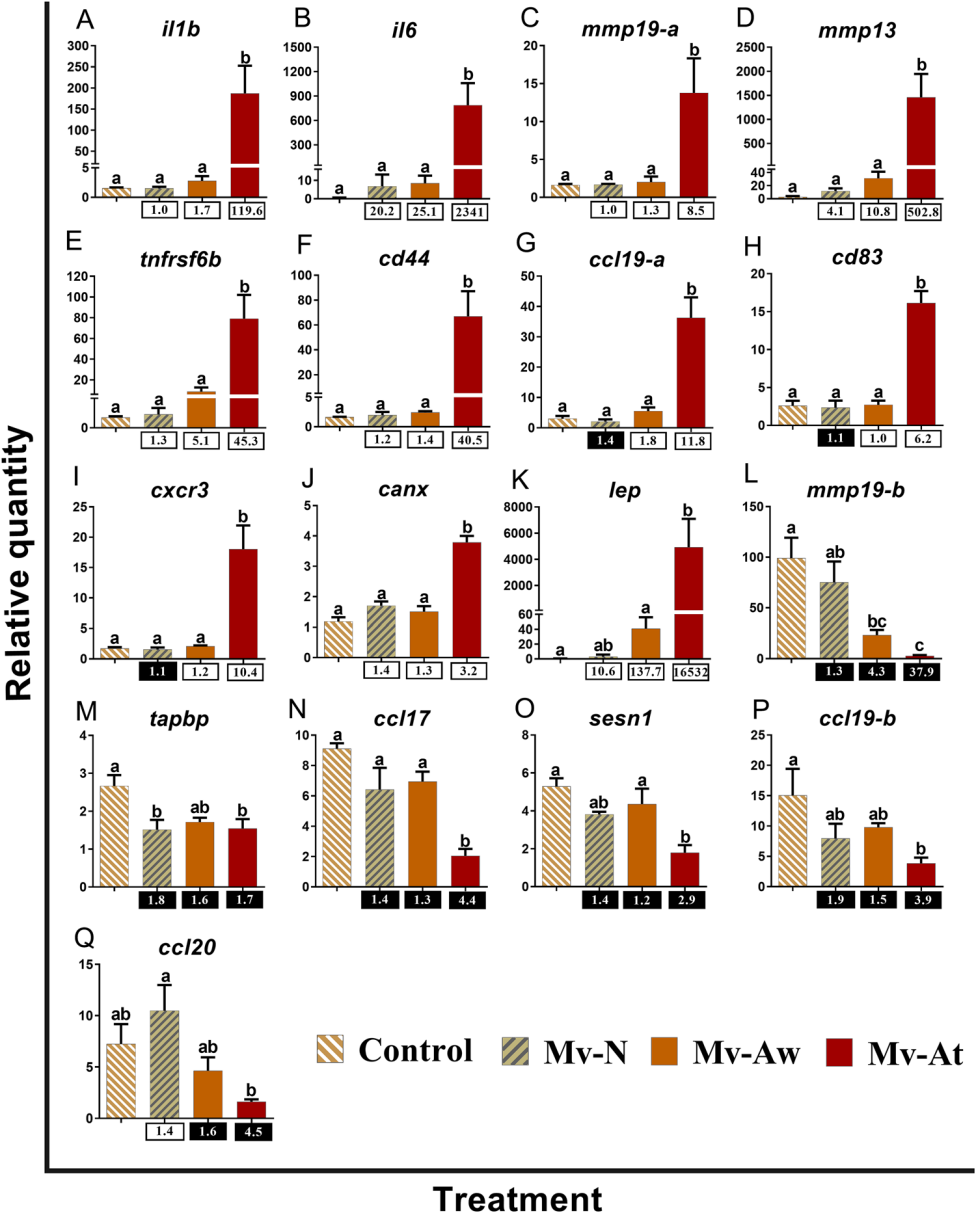
qPCR for *M. viscosa*-responsive transcripts playing putative roles in adaptive immune responses. The skin samples of *M. viscosa*-infected Atlantic salmon at the lesion (Mv-At; *n* = 4) and away (Mv-Aw; *n* = 4) sites, as well as the samples of fish with no lesion in *M. viscosa* group (Mv-N; *n* = 3) and non-infected control group (Control; *n* = 4), were used for qPCR validation. Data are presented as mean ± SE, with the lowest expressing sample as calibrator [i.e. set to relative quantity (RQ) 1.0]. Letters in each panel indicate significant differences between groups, as determined by one-way ANOVA with Tukey’s post hoc test (*p* < 0.05). Fold-changes are shown below the groups, calculated as mean RQ of the group/mean RQ of the control. Black boxes indicate the down-regulated or negative fold-changes, which were calculated as 1/fold-change for comparisons that yielded fold-change values less than 1. Gene symbols are shown at the top of each panel (**A**–**Q**).

We subjected 42 RNA-Seq-identified transcripts, with different regulatory responses to *M. viscosa* (i.e. up- and down-regulated transcripts from various lists) and various putative roles in immune processes (e.g. innate and adaptive immune responses), to reverse transcription—quantitative polymerase chain reaction (qPCR) validation. In addition to RNA-Seq studied samples (12 samples, *n* = 4), 3 skin samples from the fish that were exposed to *M. viscosa* (Mv groups) but developed no lesion or infection (Mv-N) at 29 dpi were included in the qPCR assays. All the transcripts in qPCR assays, except for *mitogen-activated protein kinase 14-paralogue a* (*mapk14-a*) in the Mv-At vs. Control comparison, showed the same fold-change direction as the RNA-Seq results (see Supplemental Table S4). Among the qPCR-studied transcripts, 22 (16 up-regulated and 6 down-regulated) transcripts were selected from the identified transcripts overlapping between Mv-At vs. Control and Mv-At vs. Mv-Aw lists (i.e. 2082 DET; Fig. 1A), and the RNA-Seq results were confirmed for all of them, except for *C–C motif chemokine 20* (*ccl20*), in at least in one of the comparisons (Supplemental Table S4). Also, nine transcripts (5 up-regulated and 4 down-regulated) were selected from the Mv-At vs. Control-specific list (i.e. 2867 DET; Fig. 1A), and significant qPCR validation (Supplemental Table S4) was seen in all but *E3 ubiquitin-protein ligase rnf213-alpha* (*rnf213a*) and *glutathione peroxidase 7* (*gpx7*). There were three qPCR-studied transcripts from the Mv-At vs. Mv-Aw-specific list (i.e. 727 DET; Fig. 1A), and only one [i.e. *mitogen-activated protein kinase 14- paralogue b* (*mapk14-b*)] of them did not show significant validation (Supplemental Table S4). Eight transcripts (4 up-regulated and 4 down-regulated) responsive to *M. viscosa* infection away site (i.e. Mv-Aw vs. Control; 563 DET; Fig. 1A) were subjected to qPCR validation, and despite showing the same fold-change direction to RNA-Seq results, significant differential expression between Mv-Aw and Control was only seen for *matrix metalloproteinase-19-paralogue b* (*mmp19-b*) (Supplemental Table S4). Overall, there were significant correlations between qPCR and RNA-Seq results (i.e. relative quantity and normalised read counts, respectively) for 79% (i.e. 33 out of 42 transcripts) of the qPCR-studied transcripts (Supplemental Table S4). The qPCR-studied transcripts were further categorised based on their putative roles in different immune processes (Figs. 3, 4, 5).

We subjected 12 DETs with putative roles in innate immune and antibacterial responses to qPCR validation (Fig. 3). Transcript levels of *toll-like receptor 5* (*tlr5*), *C-type lectin receptor a* (*clra*), *TNF receptor-associated factor 1* (*traf1*) and *TNF receptor-associated factor 6* (*traf6*), which play roles as pattern recognition receptors (PRR) or signal transducers, were increased in the Mv-At compared to the control and Mv-Aw groups (Fig. 3A–D). Transcription factors, *interferon regulatory factor 7* (*irf7*) and *cAMP-responsive element modulator* (*crem*), were up-regulated in the lesion site of *M. viscosa* infection (i.e. Mv-At), compared to the other groups (Fig. 3E,F). A similar transcript expression profile was seen for *CC chemokine* (*ccl*), *hepcidin antimicrobial peptide* (*hamp*) and *cathelicidin antimicrobial peptide* (*camp*) (Fig. 3G–I). Two paralogues of *mapk14* showed distinct responses to *M. viscosa* infection; while its paralogue* a* (*mapk14-a*) was up-regulated in the Mv-At group compared to the other groups, paralogue* b* (*mapk14-b*) up-regulation was seen in Mv-At and Mv-N compared to the control group (Fig. 3J,K). Furthermore, *M. viscosa* suppressed the expression of *activating transcription factor 2* (*atf2*) at the lesion infection site, compared with all other groups (Fig. 3L).

Thirteen DETs with putative roles in the regulation of innate immune and inflammatory responses were subjected to the qPCR validation (Fig. 4). The expression of *cholesterol 25-hydroxylase-like protein a* (*ch25ha*), *mitogen-activated protein kinase kinase kinase 8* (*map3k8*), *cold-inducible RNA-binding protein B-like* (*cirbp*) and *septin-8* (*septin8*) was significantly increased in the Mv-At group compared to the other groups (Fig. 4A–D). The levels of *integrin alpha-v* (*itgav*) were higher in the Mv-At group than those in the Control and Mv-Aw groups (Fig. 4E). Significant difference for *macrophage colony-stimulating factor 1 receptor 1* (*csf1r1*) was only seen between the Mv-At and Control groups, with higher expression in the Mv-At samples (Fig. 4F). *E3 ubiquitin-protein ligase rnf213-alpha* (*rnf213a*) expression significantly increased in the Mv-At compared with the Mv-N group (Fig. 4G). The levels of *dual specificity mitogen-activated protein kinase kinase 4* (*map2k4*) remained unchanged in different groups (Fig. 4H). There was a significant repression for *biglycan-like* (*bgn*) by *M. viscosa* in the Mv-At and Mv-Aw samples compared to the controls (Fig. 4I). *M. viscosa* infection suppressed the expression of *NADPH oxidase 4* (*nox4*) at the skin lesion site of Atlantic salmon (Mv-At) compared to the other groups, whereas *fibroblast growth factor receptor 2* (*fgfr2*) down-regulation was only seen between the Mv-At group compared with the control and Mv-N groups (Fig. 4J,K). Although *glutathione peroxidase 7* (*gpx7*) expression was suppressed in the Mv-At samples compared to the other groups, the significant differences for this transcript were only observed between the Mv-At and Mv-N groups (Fig. 4L). No significant difference was seen among groups for *E3 ubiquitin-protein ligase RNF14* (*rnf14*) (Fig. 4M).

Seventeen transcripts involved in adaptive immune responses were subjected to qPCR validation (Fig. 5). The levels of *interleukin-1 beta* (*il1b*), *interleukin-6* (*il6*), *matrix metalloproteinase-19-paralogue a* (*mmp19-a*) and *matrix metalloproteinase-13* (*mmp13*) were significantly induced by *M. viscosa* in the Mv-At compared to the other groups (Fig. 5A–D). Similar transcript expression profiles were seen for *tumor necrosis factor receptor superfamily member 6B* (*tnfrsf6b*), *CD44 antigen* (*cd44*), *C–C motif chemokine 19-paralogue a* (*ccl19-a*), *CD83 antigen* (*cd83*), *C-X-C chemokine receptor type 3* (*cxcr3*) and *calnexin* (*canx*) (Fig. 5E–J). *M. viscosa* infection up-regulated *leptin* (*lep*) expression at the skin lesion site (Mv-At) compared to the Control and Mv-Aw groups (Fig. 5K). On the other hand, *matrix metalloproteinase-19-paralogue b* (*mmp19-b*) expression was suppressed with *M. viscosa* infection in both lesion, and away sites (i.e. Mv-At and Mv-Aw) compared to the control (Fig. 5L). There were lower levels of *tapasin* (*tapbp*) in Mv-N and Mv-At groups than in the control group (Fig. 5M). *C–C motif chemokine 17* (*ccl17*) and *sestrin-1* (*sesn1*) were significantly down-regulated in response to *M. viscosa* infection at the lesion site compared to the Mv-Aw and control samples (Fig. 5N,O). *C–C motif chemokine 19-paralogue b* (*ccl19-b*) expression was significantly suppressed in Mv-At compared with control samples (Fig. 5P). The significant down-regulation of *C–C motif chemokine 20* (*ccl20*) by *M. viscosa* infection was only seen between the Mv-At and Mv-N groups (Fig. 5Q).

**Figure 6:**
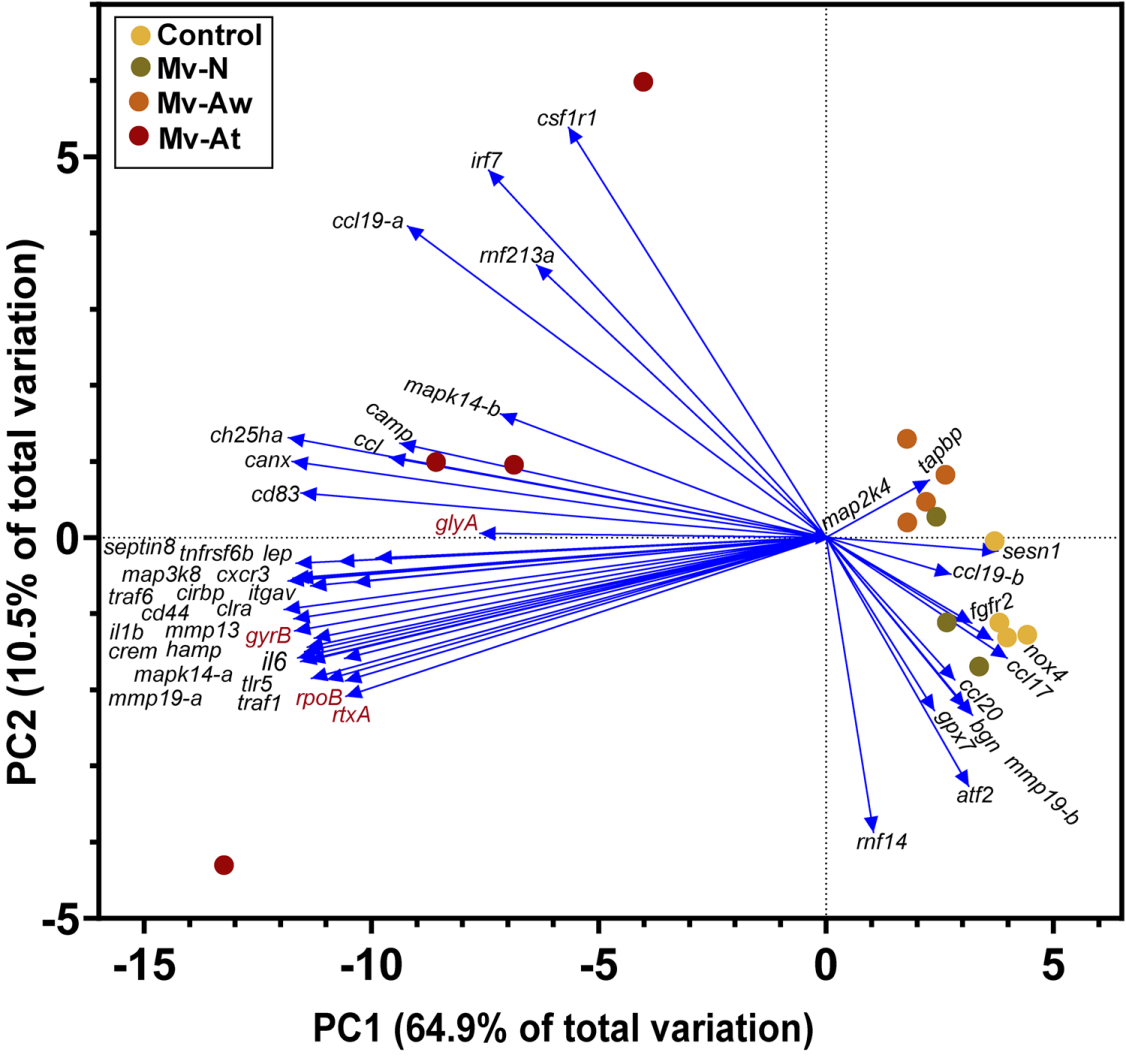
Principal Component Analysis (PCA) using all qPCR-studied transcripts for Atlantic salmon and *M. viscosa*. The skin samples of *M. viscosa*-infected Atlantic salmon at the lesion (Mv-At; *n* = 4) and away (Mv-Aw; *n* = 4) sites, as well as the samples of fish with no lesion in *M. viscosa* group (Mv-N; *n* = 3) and non-infected control group (Control; *n* = 4), were used for qPCR. Atlantic salmon and *M. viscosa* transcripts are shown in black and red, respectively.

Principal component analysis (PCA) was performed to examine the association of Atlantic salmon and *M. viscosa* transcripts studied herein, and it exhibited a clear separation among the experimental groups (Fig. 6). PC1 (i.e. 64.9% of total variation) and PC2 (i.e. 10.5% of total variation) cumulatively explained 75.4% of the total variation. The Control, Mv-Aw and Mv-N samples were positively loaded on PC1, whereas Mv-At samples were negatively loaded on PC1 (Fig. 6). While the lesion site infection samples (i.e. Mv-At) associated with the left side vectors such as *crem*, *irf7*, *tlr5*, *csf1r1* and *ch25ha* as well as the *M. viscosa* transcripts (i.e. *glyA, rpoB, rtxA* and *gyrB*), samples from the other groups (i.e. Mv-Aw, Mv-N and Control) were associated with the right side vectors (e.g. *tapbp*, *ccl20* and *atf2*).

## Discussion

This was the first study profiling the transcriptome response of Atlantic salmon skin to *M. viscosa,* the causative agent of winter-ulcer disease. Samples from the skin of *M. viscosa*-infected Atlantic salmon at the lesion and away sites as well as the control group, collected at 29 days post-infection, were used for RNA-Seq analyses. Although the number of acquired reads for each sample varied between ~ 40 to 87 million among samples, the proportions of filtered and mapped reads (e.g. ~ 94–96% overall mapping) for all the samples used in the current study were comparable, reflecting the consistency of RNA-Seq procedures. Therefore, the identified DETs in this study arose from the experimental conditions, and were not influenced by technical variations. The present study found 5212 and 2911 DETs in response to *M. viscosa* for the Mv-At vs. Control and Mv-At vs. Mv-Aw comparisons, respectively, and 2105 of these DETs overlapped between two comparisons. Moreover, 563 DETs was identified comparing the Mv-Aw with the control group, 23 of which overlapped with other DETs in other transcript lists (Fig. 1A). The hierarchical analysis showed that all the samples associated with a given experiential group were clustered together, reflecting the comparable gene expression profiles of samples in each group. An RNA-Seq based study identified 8580 differentially expressed genes in the skin of *Aeromonas hydrophila*-infected goldfish (*Carassius auratus*) at 12 h post-infection^31^. Likewise, our global gene expression results showed a large number of DETs in Atlantic salmon skin infected with *M. viscosa*. Although there are few studies focusing on the transcriptome response of fish skin to bacterial infections, various studies profiled fish skin and fin transcriptome responses to parasitic pathogens. RNA-Seq analyses identified 4355 responsive genes in sea lice (*Caligus rogercresseyi*) attachment sites of Atlantic salmon skin, compared to the healthy skin^32^. A microarray-based study identified 2239 and 6227 differentially expressed probes (DEPs) in sea lice (*Lepeophtheirus salmonis*) attachment and adjacent fin sites, respectively, compared to the control, which suggests stronger transcriptional dysregulation in Atlantic salmon skin adjacent to (i.e. away from) sea lice attachment^33^. In contrast to the sea lice-driven gene dysregulation^33^, our findings showed that *M. viscosa* caused a massive gene dysregulation at the lesion site of Atlantic salmon skin, but it had more subtle effects on the skin transcriptome of infected fish beyond the lesion site (i.e. 5212 DETs vs. 563 DETs; Fig. 1A). Further, as found by clustering analysis (Fig. 1B), the transcriptome profiles of the away site samples (Mv-Aw) showed more resemblance to the control samples than those of the lesion site samples. Collectively, these results suggest that the *M. viscosa*-triggered strong immune response of Atlantic salmon skin may be restricted largely to the lesion site (e.g. 2867 DETs specific to Mv-At), and the magnitude of host transcriptional responses to this pathogen remarkably lower beyond the infection site.

To validate the RNA-Seq results, 42 transcripts from all the comparisons and with a varying magnitude of response (i.e. up- and down-regulated transcripts) and putative immunological roles (e.g. innate and adaptive immunity) were subjected to qPCR analyses, and all of them, except for *mapk14-a*, showed the same fold-change directions as the RNA-Seq results. Also, there were variations between RNA-Seq and qPCR results for the significant differences found among treatments (Supplemental Table S4). The variations seen between the RNA-Seq and qPCR analyses may result from differences in sensitivity of these methods for assessing gene expression. Furthermore, differences in statistical significance for a given comparison between qPCR and RNA-Seq may be attributed to the variations in the distribution of acquired values (i.e., normalised fluorescence ratios vs. normalised read counts, respectively) as well as stringency level and statistical methods (i.e. DESeq2 vs. One-way ANOVA, respectively) used for data analyses. In general, however, the qPCR data showed a very high degree of agreement with the RNA-Seq results (e.g. significant correlation between the qPCR and RNA-Seq results for 79% of transcripts), which indicates the robustness of the transcriptome data presented herein.

**Figure 7:**
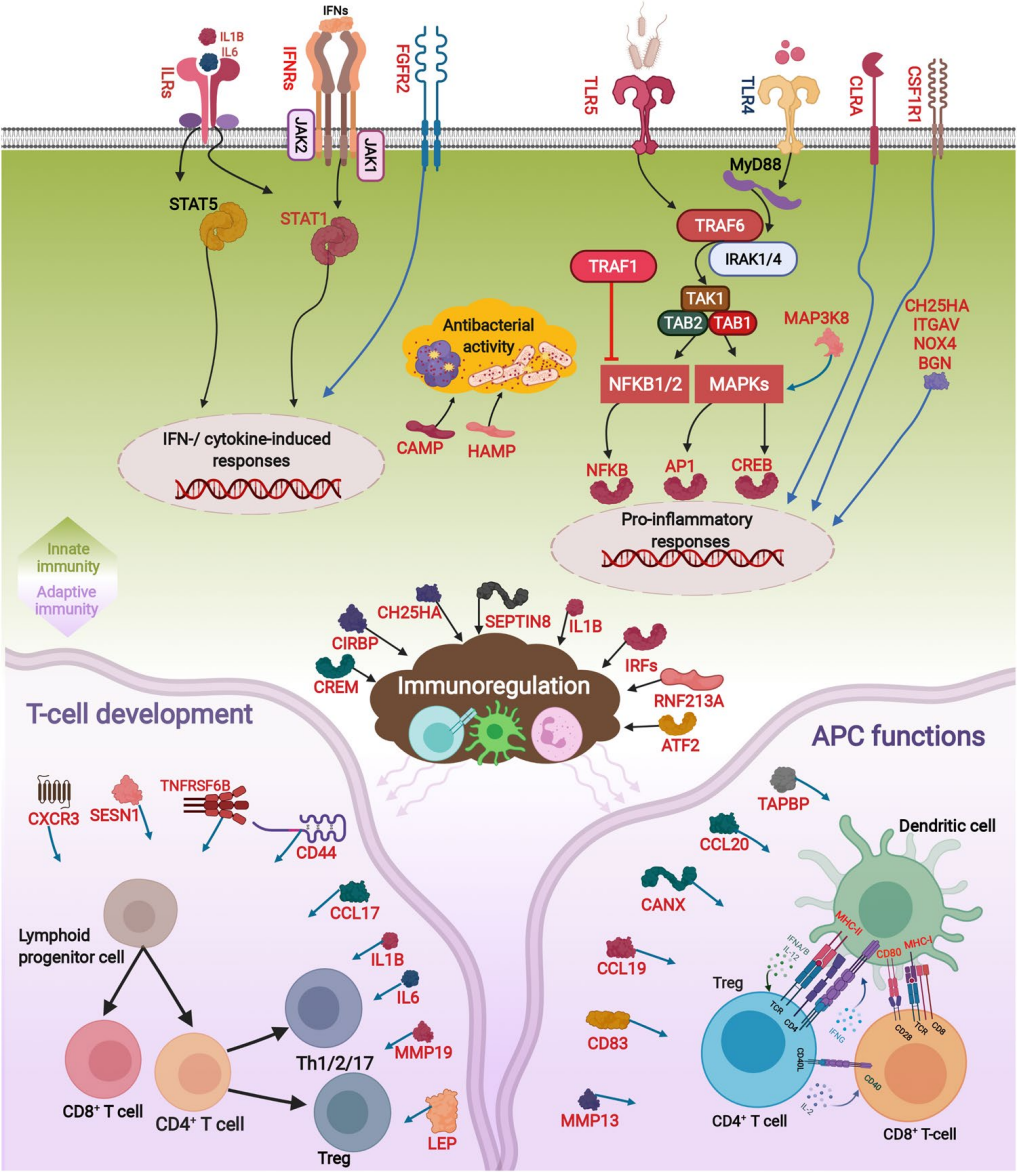
The adaptive and innate immune pathways dysregulated by *M. viscosa* infection in Atlantic salmon skin. The figure, generated using BioRender (https://biorender.com/), was developed using the identified genes in the present study and their known functions in mammals, as explained in the Discussion section. The *M. viscosa*-responsive genes are shown in red font or red boxes. Black and blue arrows indicate activatory and regulatory effects, respectively, whereas an inhibitory effect is shown with red line. Clouds and oval circles reflect biological processes and gene expression activation, respectively. Toll-like receptor (TLR), Myeloid differentiation primary response 88 (MyD88), Tumor necrosis factor (TNF), TNF receptor-associated factor (TRAF), Interleukin-1 receptor-associated kinase (IRAK), Transforming growth factor beta (TGFB)-activated kinase 1 (TAK1), TAK1-binding protein (TAB), Nuclear factor kappa-B (NFKB), Mitogen-activated protein kinase (MAPK), Transcription factor AP1 (AP1), cAMP response element-binding protein (CREB), Interleukins (ILs), Interferons (IFNs), IFN receptors (IFNRs), IL receptors (ILRs), Janus kinase (JAK), Tyrosine kinase (TYK), Signal transducer and activator of transcription (STAT), Interferon regulatory factor (IRF), Hepcidin antimicrobial peptide (HAMP), Cathelicidin antimicrobial peptide (CAMP), Cholesterol 25-hydroxylase-like protein A (CH25HA), NADPH oxidase 4 (NOX4), Biglycan-like (BGN), Mitogen-activated protein kinase kinase kinase 8 (MAP3K8), E3 ubiquitin-protein ligase RNF213-alpha (RNF213A), Macrophage colony-stimulating factor 1 receptor 1 (CSF1R1), C-X-C chemokine receptor type 3 (CXCR3), Calnexin (CANX), Leptin (LEP), Tapasin (TAPBP), Sestrin-1 (SESN1), CD antigen (CD), C-type lectin receptor a (CLRA), cAMP-responsive element modulator (CREM), Cold-inducible RNA-binding protein B-like (CIRBP), Septin-8 (SEPTIN8), Fibroblast growth factor receptor 2 (FGFR2), Matrix metalloproteinase (MMP), T-cell receptor (TCR), Major histocompatibility complex I/II (MHC-I/II), Tumor necrosis factor receptor superfamily member 6b (TNFRSF6B), C–C motif chemokine (CCL19), Activating transcription factor 2 (ATF2), Integrin alpha-v (ITGAV), T helper 1/2/17 cell (Th1/2/17), regulatory T-cells (Treg), antigen-presenting cell (APC).

The RNA-Seq results found *M. viscosa-*dependent dysregulation of several transcripts involved in innate immunity, and our qPCR confirmed the induction of transcripts encoding innate immunity-relevant PRRs (*tlr5* and *clra*), signal transducers (*traf6, traf1* and *mapk14-a*), transcription factors (*irf7, crem*) and immune effectors (*ccl, hamp* and *camp* ). There was a close association between the expression patterns of these transcripts with the expression of *M. viscosa* housekeeping (*rpoB*, *glyA* and *gyrB*) and virulence (*rtx-A*) genes (Fig. 6). Mammalian TLR5 is involved in the recognition of bacterial flagellin, leading to the Myeloid differentiation primary response 88 (MyD88)-dependent pathway activation^34^, and *tlr5* induction in our study may be linked to *M. viscosa* flagellin-driven responses^12^. Figure 7 illustrates the putative immune pathway activated by *M. viscosa* in Atlantic salmon skin. In addition, *M. viscosa* changed the expression of the MyD88-associated factors (i.e. *traf6, traf1* and *irf7*) that are known to promote and/or regulate the activity of Nuclear factor kappa B (NFKB), and thereby the production of inflammatory cytokines^35,36^. *M. viscosa* dysregulated NFKB- and MAPK-dependent (e.g. *nfkb*, *ap1*, *creb*, *mapk14-a* and *atf2*) and other (e.g. *irf7* and *crem*) transcription factors which play a central role in the regulation of immune responses and production of IFNs, ILs and other immune effectors^35,37–39^. Accordingly, activation of cytokine-dependent pathways (i.e. type I and II IFNs, and IL pathways) involved in secondary immune responses was found herein following *M. viscosa* infection (Fig. 7). *M. viscosa* strongly induced *camp* and *hamp*, shown to have bactericidal activities in teleosts (e.g. rainbow trout)^40–43^, and it reflects the activation of IFN/TLR-inducible antibacterial processes in Atlantic salmon skin in response to the winter-ulcer disease.

We observed *M. viscosa*-dependent dysregulation for a large array of transcripts involved in immunoregulatory processes. *M. viscosa* induced *ch25ha, map3k8, cirbp, septin8, itgav, csf1r1* and *rnf213a,* whereas the expression of *bgn*, *nox4*, *fgfr2* and *gpx7* was suppressed in response to this Gram-negative pathogen (Fig. 4). Mammalian CH25H, MAP3K8, SEPTIN8, ITGAV and RNF213A, as well as teleost CSF1R were found to modulate inflammatory responses through regulation of immune cell activation (e.g. monocytes and neutrophils) and immune pathways (e.g. MAPK-dependent pathway)^44–49^. This is in agreement with the over-representation of immunoregulatory processes in the *M. viscosa*-induced transcript list (Supplemental Table S3A). Mammalian CIRBP was described as a novel mediator of inflammatory responses^50^, and it can contribute to the tissue damage-induced production of reactive oxygen species through CIRBP-TLR4-MyD88 signalling pathway^51^. Atlantic salmon *cirbp* was down-regulated in the liver of fish exposed to elevated temperature^52^. In agreement with our findings, *cirbp* was induced in yellow catfish (*Pelteobagrus fulvidraco*) skin following *Edwardsiella ictaluri* infection^53^. Interestingly, a previous study established an increased adherence of *M. viscosa* to Chinook salmon embryo cell line in colder temperature (i.e. 4 °C vs. 15 °C)^12^. Further studies are needed to address if CIRBP associates with low temperature and *M. viscosa* pathogenicity. We observed up- (e.g. *septin8* and *itgav*) and down-regulation (e.g. *bgn*, *nox4*, *fgfr2* and *gpx7*) of muscle and cell structure-relevant transcripts with putative immunoregulatory roles; however, there was a higher proportion of cell structure-related BPs enriched in the *M. viscosa-*suppressed transcript list (24.3% of the terms) than those in the up-regulated transcript list (14.3% of the terms). Some of these down-regulated transcripts (e.g. *bgn* and *fgfr2*) are also associated with developmental processes, which comprised the majority (i.e. 60.1%) of BPs enriched in the *M. viscosa-*suppressed transcript list. On the other hand, "Development" only made up 12.2% of the terms enriched in the *M. viscosa-*induced transcript list (Fig. 2A). Further studies should investigate the importance of massive suppression of developmental mechanisms in the host–pathogen interactions and progression of *M. viscosa* infection. BGN and NOX4 modulate the TLR4/LPS-dependent responses^54,55^, whereas FGFR2 can change cell proliferation and survival via immune-relevant signals such as JAK/STAT^56^. GPX7 is associated with the regulation of ROS-related response in mammals^57^. The same expression pattern was seen for *gpx7* and *bgn* in Atlantic salmon skin infected with sea lice^33^, indicating the conserved regulation of these transcripts in salmon response to bacterial and parasitic infections. Nonetheless, the immune functions of these transcripts identified herein are yet to be determined for teleost species.

Our study found a massive dysregulation of the Atlantic salmon adaptive immune processes in response to *M. viscosa*, and the qPCR results confirmed the expression of 17 transcripts with putative roles in these processes. *M. viscosa* up-regulated the expression of *il1b, il6, mmp19-a, mmp13, tnfrsf6b, cd44, ccl19-a, cd83, cxcr3, canx* and *lep,* whereas levels of *mmp19-b*, *tapbp*, *ccl17, sesn1, ccl19-b* and *ccl20* were suppressed with this infection. Levels of these induced transcripts were closely associated with the expression of *M. viscosa* housekeeping (e.g. *gyrB*) and virulence (i.e. *rtx-A*) genes; however, it remains unknown if *M. viscosa* virulence factors regulate the expression of these adaptive immunity-related biomarkers identified herein. Mammalian IL1B, MMP19 and LEP play crucial roles in T-cell mediated processes such as providing cell survival signals and regulating T-cell development^58–60^. Further, mammalian IL6, TNFRSF6B, CD44, CCL17, SESN1 and CXCR3 were shown to regulate the activation, differentiation, trafficking and/or survival of helper T-cells (e.g. Th1/17)^58,61–64^. This is in parallel with the over-representation of T-cell related BPs (e.g. T-cell migration) in both *M. viscosa*-induced and -suppressed transcript lists. Likewise, our qPCR data supported dysregulation of the antigen presentation-associated transcripts. Mammalian TAPBP and CANX are involved in the Major histocompatibility class I (MHC-I) antigen presentation pathway^65^. CD83, CCL19, MMP13 and CCL20 were described to facilitate antigen-presenting cell [APC; e.g. dendritic cells (DC)]-dependent processes such as maturation, MHC stabilization, cell migration and DC-triggered T-cell activation^63,66,67^. Although the findings of the present study highlighted the importance of these APC-related transcripts in the Atlantic salmon skin immune response to *M. viscosa*, further studies are needed to better understand the function of these identified biomarkers in immune responses of Atlantic salmon and other teleosts. In the current study, two paralogues of each *mmp19* and *ccl19* showed opposite gene expression responses (i.e. induction and suppression of the paralogue *a* and *b*, respectively) to *M. viscosa*, indicating the diverged transcriptional regulation of them in Atlantic salmon antibacterial responses. Likewise, inverse regulation of *tlr5* paralogues was reported for flagellin-exposed liver cells of Japanese flounder (*Paralichthys olivaceus*)^68^. Our results suggest these adversely-regulated paralogous transcripts as suitable biomarkers for assessing the *M. viscosa*-associated responses of Atlantic salmon, yet their paralogue-specific functions remain undetermined. These paralogues may play antagonistic immune roles evolved via functional diversification of duplicate genes, as previously reported for paralogues of a chemokine receptor (i.e. *cxcr3*) in zebrafish^69^.

The present study used an RNA-Seq approach to better understand the transcripts and molecular mechanisms underlying the response of Atlantic salmon to *M. viscosa* infection. *M. viscosa* elicits strong and largely site-specific gene expression responses at the lesion site, and the *M. viscosa*-triggered immune responses beyond the skin infection site are comparably subtle, i.e. fewer DETs, lower fold-change and exhibiting a gene expression profile more comparable to the control samples. RNA-Seq and qPCR results suggest that *M. viscosa* infection may activate innate immune responses through NFKB- and MAPK-dependent pathways downstream of TLR-MyD88 signals. This pathogen caused a massive dysregulation in the expression of the cytoskeleton process-related transcripts, some of which likely play roles in immune responses. Our results reflected the dysregulation of diverse adaptive immunity-associated pathways such as T-/B-cell activation, migration and antigen presentation in response to *M. viscosa*. We developed qPCR assays for several *M. viscosa*-responsive transcripts with diverse functions and regulations or paralogue-specific responses, and they can be used as valuable biomarkers for monitoring Atlantic salmon response to winter-ulcer disease and development of strategies for combating this disease. The present study draws a broad picture of biological processes and molecular pathways involved in the Atlantic salmon response to *M. viscosa*. However, further studies are required to characterise the functions of the *M. viscosa*-responsive transcripts identified herein.

## Materials and methods

### Animals

All procedures in the present study were performed under approval of the Animal Care Committee of the University of Prince Edward Island (UPEI). Unvaccinated Atlantic salmon [214.6 ± 41.44 g (mean ± SD)] smolts were obtained from Cooke Aquaculture, New Brunswick, Canada, and transported to the Aquatic Animal Facility (biological containment level II) in the Atlantic Veterinary College (AVC) at UPEI.

Fish were distributed to 4 circular tanks (350 L tanks; 40 fish per tank), using seawater recirculating systems (water flow: ~ 8 L min^−1^). Fish were fed at 1–1.5% of the body weight daily using a standard EWOS commercial diet (Dynamic S^®^) at 10 °C and under 12 h light and 12 h dark photoperiod. To prevent transmission of bacteria between groups, fish in control (2 tanks) and *M. viscosa* groups (2 tanks) were kept in different rooms, connected to separate recirculating systems. Fish were acclimatised to the experimental conditions three weeks prior to the *M. viscosa* challenge, and water quality parameters were monitored daily during the experiment. Fish were fasted 24 h before all experimental procedures.

### *Moritella viscosa* strain and culture

*Moritella viscosa* culture was prepared from a stock of FFA-371, a variant clade Pacific Ocean isolate from British Columbia, Canada, at the New Brunswick Research and Productivity Council (RPC; Fredericton, NB, Canada). Cultures were grown in Tryptone Soya Broth with a final sodium chloride concentration of 2% and incubated at 8.0 ± 1.0 °C with agitation.

### *Moritella viscosa* infection

One week prior to *M. viscosa* infection, the water temperature in all tanks was lowered from 10.0 ± 1.0 °C to 8.0 ± 1.0 °C and maintained at this temperature for the remainder of the trial. Fish in both experimental and control tanks were fasted 24 h before and after the infection challenge. Fish in *M. viscosa* group (i.e. two tanks per treatment, 40 fish per tank) were infected with *M. viscosa,* whereas control fish were exposed to a sham infection (i.e. using 40 mL of sterile seawater). The immersion exposure system consisted of 200 L tanks equipped with airstones, and 40 mL of bacterial seawater suspension of *M. viscosa* or sterile seawater were added to the exposure tanks of infection or control (sham infection) groups, respectively. The final concentration of *M. viscosa* in challenge tanks was 1.0 × 10^5^ colony-forming units (cfu) mL^−1^. The exposure tanks were not connected to the recirculating systems. Fish from a given tank were transferred to the corresponding exposure tanks (*M. viscosa* or sham infection tanks), and fish welfare (e.g. normal swimming or no sign of stress) was checked every 5 min during the challenge. After 60 min of immersion exposure to *M. viscosa* or sham infection (control), fish were transferred to the original tanks. Fish were kept at 8.0 ± 1.0 °C and fed as described above and the water quality parameters (i.e. oxygen saturation: ~ 100%, ammonia–nitrogen: 0.00–0.05 mg L^−1^, nitrite-nitrogen: 0.00–0.15 mg L^−1^, nitrate-nitrogen: 0–60 mg L^−1^, pH: 7.8–8.5 and salinity 33–36 ppt) and fish mortality were monitored daily.

### Sampling and RNA extraction

The skin samples of fish were collected at 29 days post infection. Four fish in each tank (i.e. *n* = 8) were taken opportunistically and euthanized using 300 mg L^−1^ of MS222 (Syndel Laboratories, Vancouver, BC, Canada). In the control group, the skin samples were collected from a specific site (i.e. 1 cm above the anal fin on the left side skin) from all fish. The skin samples at the lesion sites (at the edge of lesion: Mv-At) and away sites (~ 1 cm away from the edge of the lesion; Mv-Aw) were taken from fish that developed skin lesions (i.e. stage 3 lesion, which includes dermal ulceration along with visible muscle tissue^70^; 5 out of the 8 sampled fish had lesions) in the *M. viscosa* group, whereas fish with no lesion (3 out of the 8 sampled fish did not have lesions; Mv-N) in the *M. viscosa* group were sampled as in fish from the control group. Supplemental Fig. S2 shows a stage 3 lesion in the skin of *M. viscosa*-infected Atlantic salmon as well as the lesion and away sampling sites.

Total RNA of skin samples was extracted using TRIzol (Invitrogen, Thermo Fisher Scientific, Waltham, MA, USA) following the manufacturer's instructions. Samples were homogenised in 400 µl TRIzol using 5 mm stainless steel beads (Qiagen, Hilden, Germany) using a TissueLyser LT (Qiagen) for 3 min. An additional 400 µl TRIzol was added to each sample and samples were further homogenised for 3 min. The TRIzol-lysed samples were then passed through QIAshredder (Qiagen) homogenizer spin columns prior to the RNA extraction. Following the extraction of the crude RNA, RNA samples (~ 40 µg) were treated with 6.8 Kunitz units of DNaseI (Qiagen) for 10 min at room temperature to remove residual genomic DNA. DNase-treated RNAs were column-purified using the RNeasy MinElute Cleanup Kit (Qiagen) following the manufacturer’s instructions. The concentration and integrity of column-purified RNAs were determined using NanoDrop spectrophotometry (ND-1000) and 1% agarose gel electrophoresis, respectively. The RNA samples used in the present study showed high purity (i.e., A260/230 and A260/280 ratios > 1.8) and integrity (i.e., tight 18S and 28S ribosomal RNA bands).

### Sample selection for transcriptome analyses

We measured the expression of *M. viscosa*-specific genes^16,18^ as well as host immune response biomarkers to select a subset of samples for transcriptome analyses. Among *M. viscosa*-specific genes studied herein, *serine hydroxymethyltransferase* (*glyA*), *RNA polymerase* (*rpoB)* and *DNA gyrase subunit B* (*gyrB*) play roles as housekeeping genes^16,18^, whereas *repeats in toxin* (*rtx-A)* is a putative virulence factor^18^. qPCR assays were performed as described in the qPCR validation section. Samples (i.e. one Mv-At and one Mv-Aw) from one fish (i.e. 1 out of the 5 sampled fish with lesions) in the *M. viscosa* group were not included in transcriptome analyses as they failed RNA extraction. All Mv-At samples from the 4 fish with lesions in the infection group, showed expression of *M. viscosa*-specific genes (data not shown) as well as strong induction of assessed antibacterial biomarkers [i.e. *hamp*, *camp* and *il1b* up-regulation in the Mv-At compared to the other groups (Figs. 3H,I and 5A]. The expression of *M. viscosa*-specific genes was not detected in the Control and Mv-N samples as well as 3 out 4 Mv-Aw samples (data not shown). In addition, there was no significant difference between the control, Mv-Aw and Mv-N groups in expression of immune response biomarkers [i.e. *hamp*, *camp* (Fig. 3H,I) and *il1b* (Fig. 5A). Therefore, to profile the transcriptome response of Atlantic salmon to *M. viscosa,* the lesion (Mv-At: *n* = 4) and away (Mv-Aw: *n* = 4) samples from 4 fish (i.e. showing expression of *M. viscosa* genes in all Mv-At samples) with stage 3 lesion (i.e. dermal ulceration with visible muscle tissue^70^) as well as 4 fish from the control group were selected for RNA-Seq analyses (12 samples in total).

### RNA-Seq analysis

Library construction and RNA sequencing services in the present study were provided by the McGill University and Génome Québec Innovation Centre. The quality of RNAs was assessed using Bioanalyzer 2100 (Agilent, Santa Clara, CA, USA), and all of the samples showed high RNA integrity and no degradation [i.e. RNA Integrity Number (RIN): 9.8-10]. The library construction of the 12 samples used in the current study was performed NEBNext mRNA Library Prep Reagent Set for Illumina (New England Biolabs, Ipswich, MA, USA), following the manufacturer’s instructions. The sequencing was conducted using Illumina NovaSeq 6000 [S2, paired-end 100 bp (PE100)-50 M reads]. Supplemental Table S1 shows the summary for RNA-Seq and library construction of 12 samples in the present study.

### RNA-Seq data processing

The quality control assessment, trimming, and filtering low-quality reads were performed using FastQC v0.11.8 and Trimmomatic v0.39^71^. We used HISAT2 v2.1.0, a rapid and accurate alignment tool for mapping sequencing reads to the Atlantic salmon genome (version: GCF_000233375.1_ICSASG_v2). StringTie v2.0 was used to assemble and calculate the expression levels of all the transcripts of the aligned reads using reference gene models provided as annotation files (i.e. GTF files) that are available and distributed together with the Atlantic salmon genome. We also used StringTie to assemble and quantify novel genes and transcripts. The accuracy of transcript assembly was evaluated using the gffcompare v0.11.2 program. The read count data used for the differential expression analysis were obtained from python script ‘prepDE.py’ provided by StringTie authors (http://ccb.jhu.edu/software/stringtie/dl/prepDE.py3). The differential expression analysis was performed using DESeq2 in Bioconductor package (DEseq2 v1.28.1) and transcripts with expression [TPM (transcripts per million) > 1] in at least two replicates of each group at adjusted *p* value < 0.01. Since the present study is the first report on transcriptome responses of Atlantic salmon skin to winter-ulcer disease, annotation and differential expression analyses were conducted at the transcript level to provide a more comprehensive understanding of the transcriptional regulations (e.g. post-transcriptional processes and transcript variants) of Atlantic salmon in face of *M. viscosa* infection. The identified DETs were re-annotated using the BLASTx searches of NCBI’s non-redundant (nr) amino acid sequence (E-value < 1e−05), as implemented by Blast2GO software (BioBam Bioinformatics S.L., Valencia, Spain)^72,73^. The heatmap of identified DETs was generated, using heatmap3 function of gplots package (version 3.1.1) in R. The pathway analyses were performed, as explained in Eslamloo et al.^74^, to determine the enriched Biological Processes (BPs) in up- and down-regulated transcript lists of the Mv-At vs. Control and Mv-At vs. Mv-Aw comparisons. The enrichment (i.e. Right-sided hypergeometric test) analyses were implemented in ClueGO^75^ plugin of Cytoscape (v3.5.1)^76^, using standard human gene symbols of the transcripts identified herein, the Gene Ontology database (UniProt: 27.02.2019) for BPs and Benjamini–Hochberg test for *p* value corrections (*p* < 0.01). The standard human gene symbols of all transcripts in the whole RNA-Seq dataset of the present study were used as Reference for the enrichment analyses. Since the different transcript variants of a gene have the same standard gene symbol, and ClueGO conducts the analyses using the standard symbols, the enrichment analyses of the present study was necessarily performed at the gene level. The enriched BPs, identified herein, were further classified using Gene Ontology Browser (http://www.informatics.jax.org), as described in Eslamloo et al.^74^.

### qPCR validation

The qPCR assays used in this study were performed based upon the Minimum Information for Publication of qPCR Experiments (MIQE) guidelines^77^. All samples in Mv-At (*n* = 4), Mv-Aw (*n* = 4) and control (*n* = 4) groups were used for qPCR validation. In addition, samples from Mv-N groups (*n* = 3) were included in the qPCR assays. A subset of 42 RNA-Seq-identified transcripts in different comparisons was subjected to qPCR validation. The qPCR primers used in the current study were either taken from previous studies^33,78–81^ or designed using Primer3web version 4.1.0 (https://primer3.ut.ee/) (see Supplemental Table S5).

cDNA synthesis and qPCR assays (i.e. PCR program, reagents and concentrations) were performed in triplicate using a QuantStudio 5 Real-Time PCR System (384-well format) (Applied Biosystems, Thermo Fisher Scientific) as described in Eslamloo et al.^74^. Primer quality control tests [i.e. amplification efficiency^82^, a single melting peak and no amplification in the no-template control] were conducted using two cDNA pools from all samples in Mv-At and control groups for up- and down-regulated transcripts, respectively (Supplemental Table S5). The expression (i.e. C_T_ values) of 7 candidate normalisers [i.e. *60S ribosomal protein 32* (*rpl32*), *elongation factor 1 alpha-1* (*ef1a1*), *polyadenylate-binding protein, cytoplasmic 1* (*pabpc1*), *eukaryotic translation initiation factor 3 subunit D* (*eif3d*), *ATP binding cassette sub-family f member 2* (*abcf2*), *RNA polymerase 2* (*polr2*) and *NADH dehydrogenase (ubiquinone) iron-sulfur protein 7* (*ndufs7)*] in all 15 samples was analysed by geNorm, in the qBase software^83^. Two normaliser transcripts, *rpl32* and *pabpc1*, that showed low M-values (M < 0.2) and a comparable expression (i.e. C_T_ values) in all samples were used for the qPCR assays. Transcript (mRNA) levels of the transcripts of interest (TOI) and normalisers were measured as in Eslamloo et al.^74^, and the relative quantity (RQ) of each transcript was calculated using QuantStudio™ Real-Time PCR Software (Version 1.3) with incorporation of the amplification efficiencies, normalisation to both normalisers, and calibration to the sample with lowest normalised gene expression (i.e. assigned an RQ value = 1.0).

All statistical analyses were performed using the Prism package v9.0 (GraphPad Software Inc., La Jolla, CA, USA). The normality of data (i.e. RQ values) was tested using the Shapiro–Wilk normality test. One-way ANOVA was used to determine the differences among groups, and this analysis was followed by Tukey's multiple comparisons post hoc test to identify intergroup significant differences (*p* ≤ 0.05).

PCA was used to determine expression patterns among qPCR-studied TOIs of Atlantic salmon and *M. viscosa* transcripts with experimental groups. PCA was conducted in the Prism package v9.0 using standardised RQ values of qPCR data, and the selected components showed PCs with eigenvalues greater than 1.0 (Kaiser rule).

### Ethics statement

All procedures in the present study were approved (Protocol number: 18-027) by the Animal Care Committee of the University of Prince Edward Island (UPEI), following the guidelines of the Canadian Council on Animal Care. This study was performed in compliance with the ARRIVE guidelines (https://arriveguidelines.org).

## Supplementary Information

Supplementary Figures.

Supplementary Table 1.

Supplementary Table 2.

Supplementary Table 3.

Supplementary Table 4.

Supplementary Table 5.

## Data Availability

RNA-Seq data in the present study is available at Sequence Read Archive (SRA) database of NCBI (http://www.ncbi.nlm.nih.gov/sra; bioproject: PRJNA750339).
