# Supplementary material for: Global gene expression responses of Atlantic salmon skin to Moritella viscosa
Source: Sci Rep. 2022 Mar 17;12:4622. doi: 10.1038/s41598-022-08341-7 (PMC8931016; doi:10.1038/s41598-022-08341-7)
Supplement: Supplementary file 1 — Supplementary Figures. [file 41598_2022_8341_MOESM1_ESM.pdf]

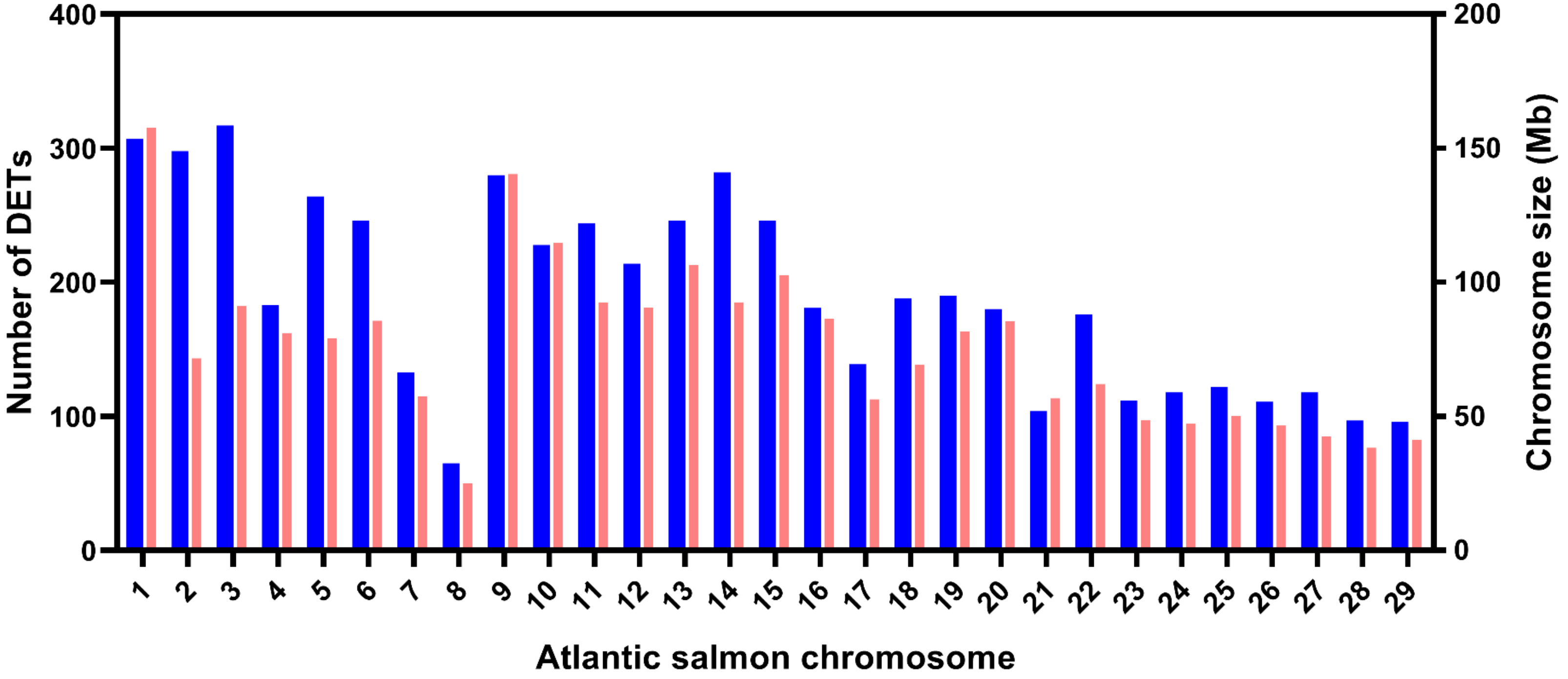


**Supplemental Figure S1.**

Chromosomal distribution of the Atlantic salmon differentially expressed transcripts (DETs) by *M. viscosa* identified in all comparisons of the present study. The remaining 754 DETs (i.e. not included in this figure) were annotated to the unplaced genomic scaffolds (i.e. 1171.9 Mb) of Atlantic salmon. Bars in azure show the number of identified DETs on each Atlantic salmon chromosome, whereas bars in rose represent the size (Mb) of each chromosome (Atlantic salmon reference genome of NCBI, version: ICSASG_v2). The genomic location of each DET is shown in Supplemental Table S2.

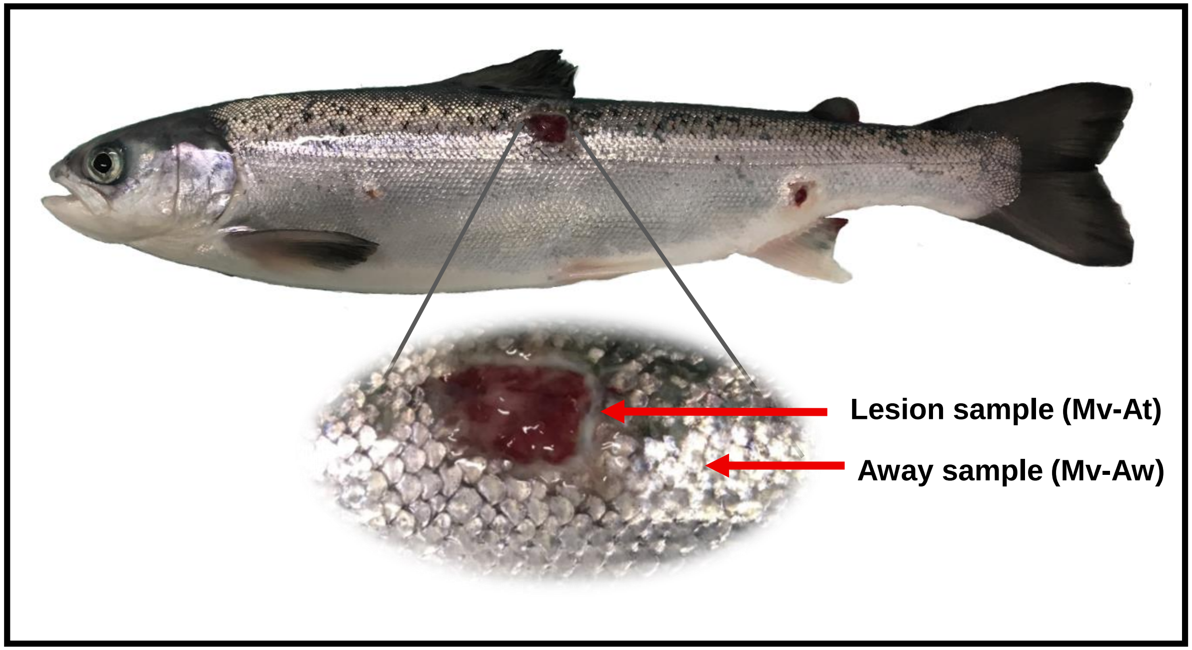


## Supplemental Figure S2.

Atlantic salmon infected with *M. viscosa* using an immersion challenge. Stage 3 lesions (i.e. magnified view; dermal ulceration and visible muscle tissue) were developed in the fish skin following 29 days of *M. viscosa* infection. The skin samples were collected from the lesion (at the edge of stage 3 lesion: Mv-At) and away (~1 cm away from the lesion edge; Mv-Aw) sites.
