# Supplementary material for: Global gene expression responses of Atlantic salmon skin to Moritella viscosa
Source: Sci Rep. 2022 Mar 17;12:4622. doi: 10.1038/s41598-022-08341-7 (PMC8931016; doi:10.1038/s41598-022-08341-7)
Supplement: Supplementary file 6 — Supplementary Table 5. [file 41598_2022_8341_MOESM6_ESM.pdf]

Supplemental Table S5. Primers used in qPCR studies

Name and symbol	GenBank accession number		Primer sequence 5' to 3'	R^2	Amplification efficiency (%)	Amplicon size (bp)
**Innate immune response**						
toll-like receptor 5 (*tlr5*)[a]	AY628755	Forward	ATCGCCCTGCAGATTTTATG	0.998	102.5	103
		Reverse	GAGCCCTCAGCGAGTTAAAG			
C-type lectin receptor a (*clra*)[a]	AY572832	Forward	CGAATCTTCAATCATGGAGAAG	0.997	94.4	117
		Reverse	TTCAGCCCCTGGGTATTTTG			
TNF receptor-associated factor 1 (*traf1*)	XM_014172399	Forward	TCGGCAGATAGACACACTGC	0.998	102.9	118
		Reverse	CTGGTTCTCCCTCTCCACAG			
TNF receptor-associated factor 6 (*traf6*)	XM_014127675	Forward	ATGTGGAGGTGATGGAGACC	0.995	96.8	104
		Reverse	CATCAGGTGCAGAAAGGTGA			
interferon regulatory factor 7 (*irf7*)[b]	FJ517644	Forward	GTCAGTGGTAAAATCAACACGC	0.999	86.5	99
		Reverse	CACCATCATGAAACGCTTGGT			
cAMP-responsive element modulator (*crem*)[c]	CB508094	Forward	GCTCTCTATGCAAGCCCTAGTC	0.995	106.4	110
		Reverse	AGACAAAATGCCCCCAGAG			
CC chemokine (*ccl*)[a]	EG850594	Forward	TTCCCTGTGTCAATGCTGTC	0.996	95.2	137
		Reverse	GGTGGTGTTCTGTGTGTCCA			
hepcidin antimicrobial peptide (*hamp*)[a]	BT125319	Forward	ATGAATCTGCCGATGCATTTC	0.996	93.8	135
		Reverse	AATGGCTTTAGTGCTGGCAG			
cathelicidin antimicrobial peptide (*camp*)[a]	GQ870278	Forward	AAGCCAGAAAATGCTCCAGA	0.998	95.9	107
		Reverse	ACCCTCAGGACGACCAATTA			
mitogen-activated protein kinase 14- paralogue a (*mapk14-a*)	AY641476	Forward	TGGCCTGTTGGATGTGTTTA	0.993	97.8	127
		Reverse	GTGGTCGTCCGTCAGTTTCT			
mitogen-activated protein kinase 14- paralogue b (*mapk14-b*)	EF123660	Forward	TTCCCGGTACTGACCACATT	0.996	90.1	102
		Reverse	TCCTTGCAGACAAAGAGGAGA			
activating transcription factor 2 (*atf2*)	XM_014173311	Forward	GTACTGCTCCTGGGTGTGGT	0.989	88.1	107
		Reverse	ACTGTTGTCCCTTGCTGGAC			
**Regulation of immune and inflammatory responses**						
cholesterol 25-hydroxylase-like protein a (*ch25ha*)[a]	BT046542	Forward	TAGAGCTGTGATGCTAGTTTAC	0.996	94.1	106
		Reverse	ACCCAGTAGCACTGAGAAGTC			
mitogen-activated protein kinase kinase kinase 8 (*map3k8*)	NM_001173785	Forward	CGCCTACCCTTCCTACCTCT	0.990	97.4[f]	110
		Reverse	TCTCCAGGGCTGTCTCTAGG			
cold-inducible RNA-binding protein B-like (*cirbp*)	XM_014148778	Forward	CTTCACTGGAAGAGGCGTTT	0.998	104.4	130
		Reverse	GCGTCTTTGGCATCTTCAG			
septin-8 (*septin8*)	XM_014197989	Forward	TCCTCTGTGTAGGGGAAACG	0.996	95.2	125
		Reverse	ATATGTTCTGGGCCGCATAC			
integrin alpha-v (*itgav*)	XM_014165693	Forward	TCAAAGCGAGTGGAAGAGGT	0.998	103.7	117
		Reverse	ACGTTTTGCTGTGGAGGAAC			
macrophage colony-stimulating factor 1 receptor 1 (*csf1r1*)[c]	CB515019	Forward	GGTTGAGGAGTTGGAGCTGT	0.997	104.6	174
		Reverse	TTGATGATGTCGGAGCTGTC			
E3 ubiquitin-protein ligase rnf213-alpha (*rnf213a*)	XM_029693039	Forward	CGTACCCTCCAAACTTCCAA	0.999	104.8	110
		Reverse	ACTCGTTCAGCGGTTCACTT			
dual specificity mitogen-activated protein kinase kinase 4 (*map2k4*)	NM_001141912	Forward	AGCCCAGCAACCAGATAATG	0.995	91.6	148
		Reverse	TCTCTGAAAAGAGCCCCGTA			
biglycan-like (*bgn*)[d]	XM_014135295	Forward	CCTTCGCATTTCTGAGGCAA	0.997	106.9	146
		Reverse	CTAGACCCAACCTGTGCAAAC			
NADPH oxidase 4 (*nox4*)	XM_014163465	Forward	AGTGGTGGGAGACTGGACTG	0.987	89.8	120
		Reverse	GGACCATCCACGTACAGCTT			
fibroblast growth factor receptor 2 (*fgfr2*)	XM_014154425	Forward	GTCCACAAACTCAGCAAGCA	0.992	95.6	146
		Reverse	TACTCTGGAATGGGGTCGTC			
glutathione peroxidase 7 (*gpx7*)	BT057727	Forward	CTTTCCGTCCTCACATTGCT	0.985	97.4	158
		Reverse	CAGTCACTGGCCACATTCAC			
E3 ubiquitin-protein ligase RNF14 (*rnf14*)	XM_014169560	Forward	TCCTCAGAGACGACCTGCTT	0.995	96.3	138
		Reverse	CTACACTGCCCTCTGCTTCC			
**Adaptive immune response**						
interleukin-1 beta (*il1b*)[e]	AY617117	Forward	GTATCCCATCACCCCATCAC	0.997	98.5	119
		Reverse	TTGAGCAGGTCCTTGTCCTT			
interleukin-6 (*il6*)	KJ425514	Forward	CCATGTTCTTCTCCAGCACA	0.996	85.7	128
		Reverse	CCTCTACCACCTCAGCAACC			
matrix metalloproteinase-19- paralogue a (*mmp19-a*)[d]	XM_014132587	Forward	CTGAACGCAGCCGTTTACT	0.996	89.9	132
		Reverse	AATATTAGGTGGGAGGCGTTTG			

matrix metalloproteinase-13 (*mmp13*)	BT046016	Forward	CTGAGACTGTGACGATGATG	0.999	98.2	108
		Reverse	AGGTAACCCGGTTGGTCTG			
tumor necrosis factor receptor superfamily member 6b (*tnfrsf6b*)[a]	EG881931	Forward	CCCAGGTCGCACCACTATAC	0.997	97.2	112
		Reverse	CATCAACTCCCCATCACAGA			
CD44 antigen (*cd44*)	XM_014148324	Forward	CCCAGTAAAGCCAACAGCAT	0.997	99.5	151
		Reverse	TTCCATCTCAGTGACCACCA			
C-C motif chemokine 19 - paralogue a (*ccl19-a*)[b]	BT125321	Forward	CTCATCAAGAAGTGCCACGA	0.999	97.5	188
		Reverse	CACCCTGTTCTTCACCCACT			
CD83 antigen (*cd83*)	BT045799	Forward	GAGCCTGTTCTGCTCATTGT	0.997	84.6	85
		Reverse	GACTCGTTGCTCAGCTTCAA			
C-X-C chemokine receptor type 3 (*cxcr3*) [c]	NM_001140493	Forward	GGTGTTGGTGCTGGTCTTTT	0.997	91.7	150
		Reverse	GCGAACGTAACCACCAGACT			
calnexin (*canx*)	XM_014199338	Forward	GGTGGTGCCTACGTCAAACT	0.997	100.7	112
		Reverse	AGTCCTCGCCACACTTGTCT			
leptin (*lep*)	XM_014124944	Forward	CCCTCCTGTTGTCCTCTCTG	0.998	99.8	156
		Reverse	CCATGCCCTCGATTAGGTTA			
matrix metalloproteinase-19- paralogue b (*mmp19-b*)	XM_014136205	Forward	CATCCCAAGAGGAAGGTTCA	0.994	102.7	159
		Reverse	TCCACAGCACAGAGATCAGG			
tapasin (*tapbp*)	BT045317	Forward	GGAGGCCACTTCTCCTCTCT	0.997	89.5	142
		Reverse	GTCTAGCCCCGCTCTCTTTT			
C-C motif chemokine 17 (*ccl17*)	XM_014216236	Forward	CATTGCCATACAGTCCATGAGAG	0.993	81.4[f]	181
		Reverse	TGTGTTTCAGGCGAGAACAAG			
C-C motif chemokine 19 - paralogue b (*ccl19-b*)[b]	BT058161	Forward	CTGCTTGACAACGACCGATA	0.994	93.1	151
		Reverse	GTTGTTCTTGGTGGCAGGAG			
C-C motif chemokine 20 (*ccl20*)	BT057039	Forward	CCAAGGAGCTGTGCAACAT	0.992	97.8	97
		Reverse	AGAGCCTTCCTCACCCATT			
sestrin-1 (*sesn1*)	XM_014205503	Forward	CTTTGCTCTCGTGGGACACT	0.990	108.5	117
		Reverse	ACATCACGAGGACGAGCTTT			
***M. viscosa* genes**						
DNA gyrase subunit B (*gyrB*)	GU124778	Forward	CGGTGTTGGTGTTTCTGTTG	0.997	103.3[f]	118
		Reverse	AGCCATTGGTCCATCTGGTA			
repeats-in-toxin A (*rtxA*)	KF822687	Forward	AGAGTGAAGAAGCCGGTCAA	0.993	105.5	122
		Reverse	ACGAAGCACATAGCCTTGCT			
DNA-directed RNA polymerase, beta-subunit (*rpoB*)	LN554852	Forward	CTGCATTAGGTCCTGGTGGT	0.995	100.6	107
		Reverse	TCCCTCTGGAGTCTCAATCG			
serine hydroxymethyltransferase (*glyA*)	LN554852	Forward	CAGCAACTAAACCTGCAACG	0.993	101.1	107
		Reverse	TTCTGCATACTCGGGTGTTG			
**Normalisers**						
60S ribosomal protein 32 (*rpl32*)	BT043656	Forward	AGGCGGTTTAAGGGTCAGAT	0.996	98.4	119
		Reverse	TCGAGCTCCTTGATGTTGTG			
polyadenylate-binding protein, cytoplasmic 1 (*pabpc1*)	EG908498	Forward	TGACCGTCTCGGGTTTTTAG	0.995	97.1	108
		Reverse	CCAAGGTGGATGAAGCTGTT			

[a] These primer sets were used in Eslamloo et al., (78)
[b] These primer sets were used in Caballero-Solares et al., (80)
[c] These primer sets were used in Eslamloo et al., (79)
[d] These primer sets were used in Umasuthan et al., (33)
[e] These primer sets were used in Zanuzzo et al., (81).
[f] The amplification efficiencies of these primers were determined using 4-point serial dilutions of cDNA.
